# Supplementary material for: Heme binding of transmembrane signaling proteins undergoing regulated intramembrane proteolysis
Source: Commun Biol. 2020 Feb 14;3:73. doi: 10.1038/s42003-020-0800-0 (PMC7021776; doi:10.1038/s42003-020-0800-0)
Supplement: Supplementary file 1 — Supplementary Information [file 42003_2020_800_MOESM1_ESM.pdf]

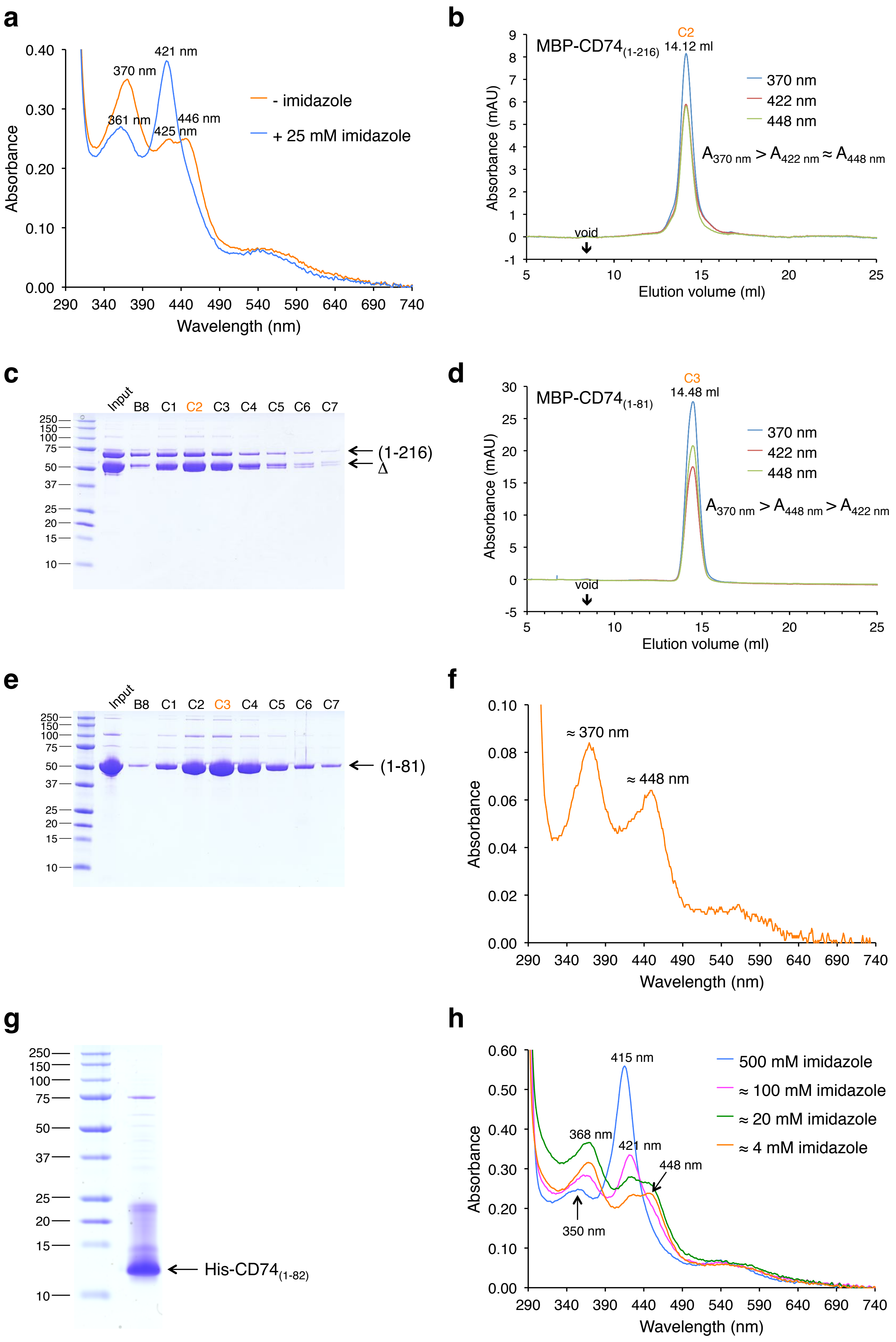

a
0.40
0.30
0.20
0.10
0.00
Absorbance
290 340 390 440 490 540 590 640 690 740
Wavelength (nm)
370 nm
421 nm
361 nm
446 nm
425 nm
- imidazole
+ 25 mM imidazole
b
MBP-CD74(1-216)
C2
14.12 ml
370 nm
422 nm
448 nm
A370 nm > A422 nm ≈ A448 nm
Absorbance (mAU)
void
Elution volume (ml)
c
Input B8 C1 C2 C3 C4 C5 C6 C7
250 150 100 75 50 37 25 20 15 10
(1-216)
Δ
d
MBP-CD74(1-81)
C3
14.48 ml
370 nm
422 nm
448 nm
A370 nm > A448 nm > A422 nm
Absorbance (mAU)
void
Elution volume (ml)
e
Input B8 C1 C2 C3 C4 C5 C6 C7
250 150 100 75 50 37 25 20 15 10
(1-81)
f
≈ 370 nm
≈ 448 nm
Absorbance
Wavelength (nm)
g
250 150 100 75 50 37 25 20 15 10
His-CD74(1-82)
h
415 nm
368 nm
421 nm
448 nm
350 nm
500 mM imidazole
≈ 100 mM imidazole
≈ 20 mM imidazole
≈ 4 mM imidazole
Absorbance
Wavelength (nm)

Supplementary Figure 1. Further characterization of heme binding by CD74. a UV/VIS spectra of MBP-CD74$_{(1-216)}$ minus (orange trace) and plus 25 mM imidazole (blue trace). **b** Purified MBP-CD74$_{(1-216)}$ containing a proteolytic degradation product was analyzed by gel filtration on a Superose 6 Increase column. Elution was followed by absorbance at 370 nm (blue trace), 422 nm (red trace) and 448 nm (green trace). MBP-CD74$_{(1-216)}$ eluted at 14.12 ml (peak fraction C2). **c** Proteins eluted between 13.0 and 16.2 ml (fractions B8 to C7; compare **b**) were analyzed by SDS-PAGE and subsequent Coomassie staining [indicated by arrows: (1-216), full length MBP-CD74 protein and Δ, degradation product]. **d** Purified MBP-CD74-NTF$_{(1-81)}$ was analyzed by gel filtration on a Superose 6 Increase column. Elution was followed by absorbance at 370 nm (blue trace), 422 nm (red trace) and 448 nm (green trace). MBP-CD74-NTF$_{(1-81)}$ eluted at 14.48 ml (peak fraction C3), whereas MBP-TNFα-NTF$_{(1-76)}$ eluted at 13.85 ml (compare Supplementary Figure 3a) and MBP-TNFα-ICD$_{(1-39)}$ at 14.83 ml (Supplementary Figure 3c). This supports our model that CD74-NTF$_{(1-81)}$ and TNFα-ICD$_{(1-39)}$ form dimers, whereas TNFα-NTF$_{(1-76)}$ forms trimers. **e** MBP-CD74-NTF$_{(1-81)}$ eluted between 13.0 and 16.2 ml (fractions B8 to C7; compare **d**) was analyzed by SDS-PAGE and subsequent Coomassie staining. **f** UV/VIS spectrum of MBP-CD74-NTF$_{(1-81)}$ purified by gel filtration (fraction C3, compare Figs. **d** and **e**). The split Soret spectrum indicated bis-thiolate coordination of ferric heme. **g** 10xHis-CD74-NTF$_{(1-82)}$ was purified by Ni-NTA from induced Lemo21(DE3) cells using Triton X100 as detergent and subsequently analyzed by SDS-PAGE. **h** UV/VIS spectroscopy of purified 10xHis-CD74-NTF$_{(1-82)}$; blue trace, after elution with 500 mM imidazole. The spectrum shows a Soret band at 415 nm indicating that two imidazole molecules instead of two cysteine residues coordinate ferric heme (analogue to a bis-histidine coordination). To restore bis-thiolate coordination of heme, 10xHis-CD74-NTF$_{(1-82)}$ was three times washed with TN buffer neither containing imidazole nor Triton X100 using Vivaspin 500 columns (Triton X100 is not removed by filtration, because of forming high molecular weight micelles). After each washing step an UV/VIS spectrum was recorded [at an imidazole concentration of about 100 mM (magenta trace), 20 mM (green trace) and 4 mM (orange trace, respectively].

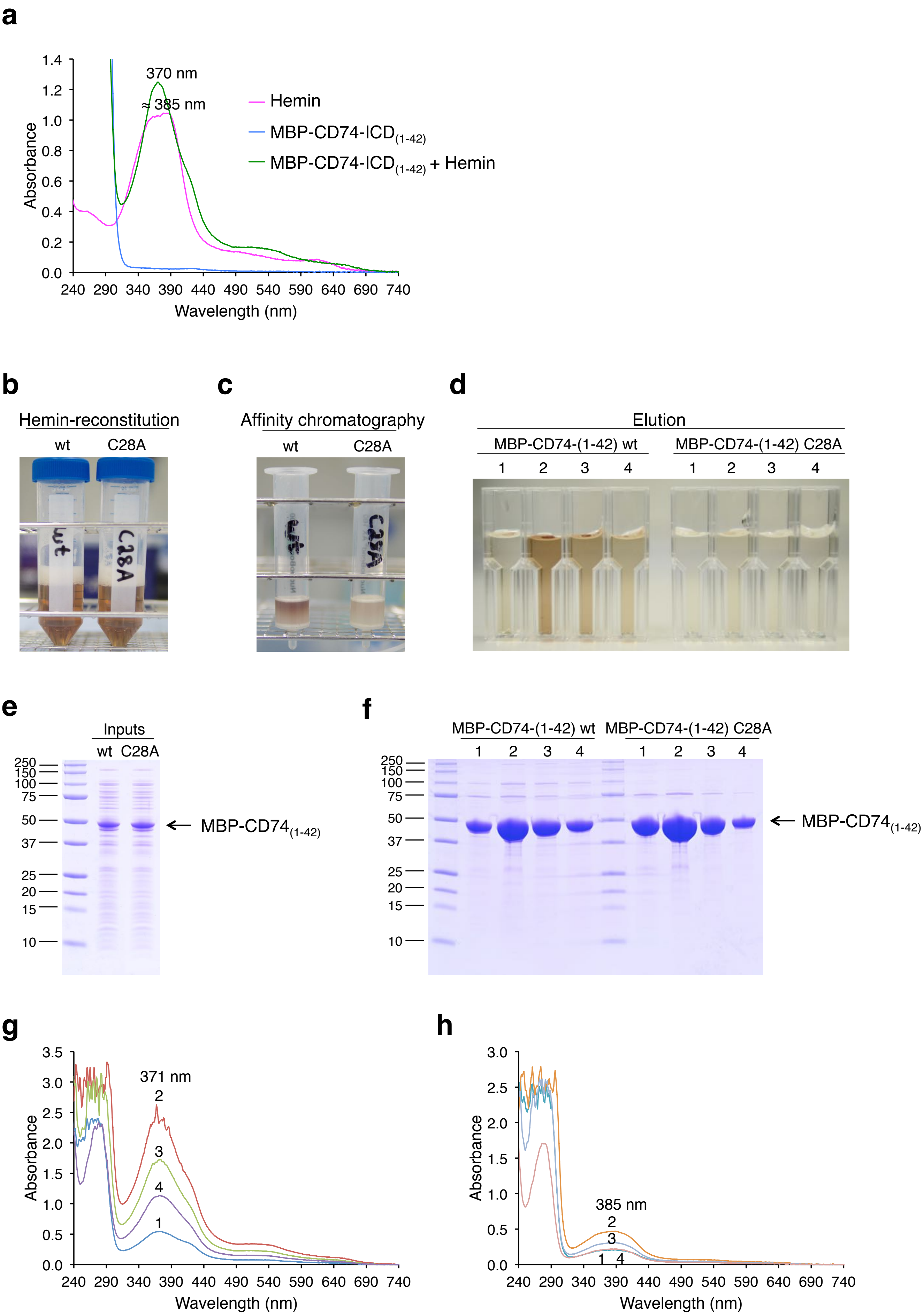
a
370 nm
≈ 385 nm
Hemin
MBP-CD74-ICD(1-42)
MBP-CD74-ICD(1-42) + Hemin
Absorbance
Wavelength (nm)
b
Hemin-reconstitution
wt
C28A
c
Affinity chromatography
wt
C28A
d
Elution
MBP-CD74-(1-42) wt
MBP-CD74-(1-42) C28A
1 2 3 4
1 2 3 4
e
Inputs
wt C28A
250 150 100 75 50 37 25 20 15 10
MBP-CD74(1-42)
f
MBP-CD74-(1-42) wt
MBP-CD74-(1-42) C28A
1 2 3 4
1 2 3 4
250 150 100 75 50 37 25 20 15 10
MBP-CD74(1-42)
g
371 nm
Absorbance
Wavelength (nm)
h
385 nm
Absorbance
Wavelength (nm)

Supplementary Figure 2. Hemin reconstitution of CD74-ICD$_{(1-42)}$. a Hemin reconstitution of amylose purified MBP-CD74-ICD$_{(1-42)}$ was shown by UV/VIS spectroscopy. Magenta trace, 25 μM hemin in elution buffer (10 mM maltose in TN-buffer); blue trace, 56 μM MBP-CD74-ICD$_{(1-42)}$ in elution buffer and green trace, 56 μM MBP-CD74-ICD$_{(1-42)}$ plus 25 μM hemin in elution buffer. **b** Cytosolic protein solutions of *malE-CD74-ICD-(1-42) wt* and *malE-CD74-ICD-(1-42) C28A*, respectively, expressing *E. coli* cells were reconstituted with 50 μM hemin before amylose affinity chromatography. **c** Amylose affinity chromatography of hemin-reconstituted MBP-CD74-ICD$_{(1-42)}$ wt and C28A fusion proteins; amylose columns are shown after washing with TN-buffer. **d** Eluted MBP-CD74-ICD$_{(1-42)}$ fusion proteins were collected in four fractions. **e** Inputs and **f** proteins eluted from the amylose columns were analyzed by SDS-PAGE. **g** Elution of MBP-CD74-ICD$_{(1-42)}$ wt and **h** MBP-CD74-ICD$_{(1-42)}$ C28A proteins in four fractions (different colored traces 1 to 4) was followed by UV/VIS spectroscopy. The absorbance maximum at 371 nm of wt protein indicated that ferric heme is high-spin, penta-coordinate and with a cysteine thiolate as the fifth ligand.

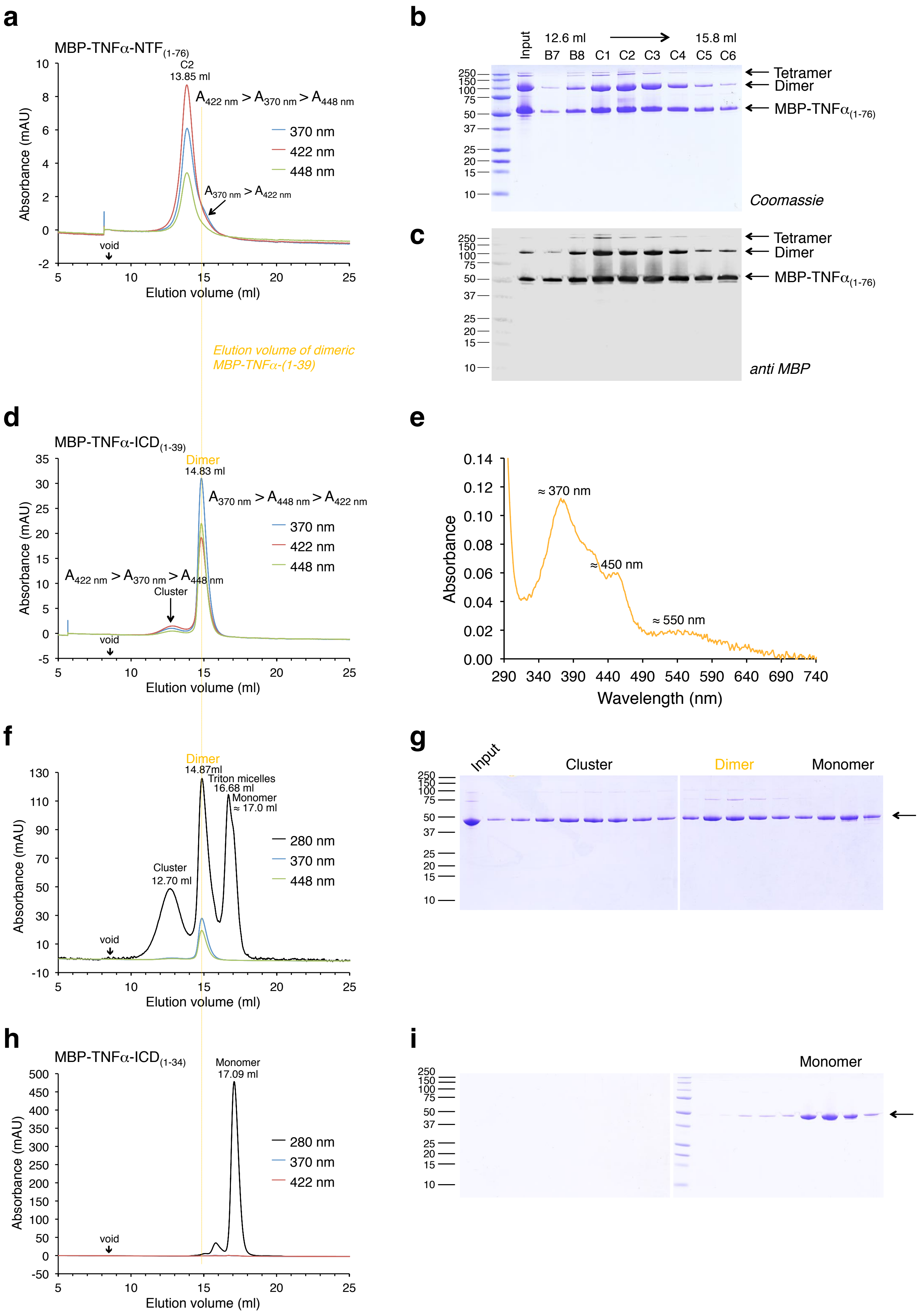
a
MBP-TNFα-NTF(1-76)
C2
13.85 ml
A422 nm > A370 nm > A448 nm
A370 nm > A422 nm
370 nm
422 nm
448 nm
Absorbance (mAU)
void
Elution volume (ml)
b
Input
12.6 ml
15.8 ml
B7 B8 C1 C2 C3 C4 C5 C6
Tetramer
Dimer
MBP-TNFα(1-76)
Coomassie
c
Tetramer
Dimer
MBP-TNFα(1-76)
anti MBP
Elution volume of dimeric MBP-TNFα-(1-39)
d
MBP-TNFα-ICD(1-39)
Dimer
14.83 ml
A370 nm > A448 nm > A422 nm
A422 nm > A370 nm > A448 nm
Cluster
370 nm
422 nm
448 nm
Absorbance (mAU)
void
Elution volume (ml)
e
≈ 370 nm
≈ 450 nm
≈ 550 nm
Absorbance
Wavelength (nm)
f
Dimer
14.87ml
Triton micelles
16.68 ml
Monomer
≈ 17.0 ml
Cluster
12.70 ml
280 nm
370 nm
448 nm
Absorbance (mAU)
void
Elution volume (ml)
g
Input
Cluster
Dimer
Monomer
h
MBP-TNFα-ICD(1-34)
Monomer
17.09 ml
280 nm
370 nm
422 nm
Absorbance (mAU)
void
Elution volume (ml)
i
Monomer

Supplementary Figure 3. Gel filtration of MBP-TNFα-NTF$_{(1-76)}$, MBP-TNFα-ICD$_{(1-39)}$, and MBP-TNFα-ICD$_{(1-34)}$. a Purified MBP-TNFα-NTF$_{(1-76)}$ was analyzed by gel filtration on a Superose 6 Increase column. Elution was followed by absorbance at 370 nm (blue trace), 422 nm (red trace) and 448 nm (green trace). Elution of MBP-TNFα-NTF$_{(1-76)}$ between 12.6 ml and 15.8 ml (fractions B7 to C6) from the Superose 6 column was analyzed by **b** SDS-PAGE and Coomassie staining and **c** by immunoblotting with anti MBP antibody. SDS-stable oligomers may occur because of the Fenton reaction (formation of radicals in presence of iron ions leads to covalent linkage of heme coordinating monomers), SDS-stable dimers may also be explained by disulfide bond formation. **d** MBP-TNFα-ICD$_{(1-39)}$ purified from the membrane fraction with Triton X100 was analyzed by gel filtration on a Superose 6 Increase column. Elution was followed by absorbance at 370 nm (blue trace), 422 nm (red trace) and 448 nm (green trace). **e** MBP-TNFα-ICD$_{(1-39)}$ eluted between 14.6 and 15.0 ml from the Superose 6 Increase column was characterized by UV/VIS spectroscopy. **f** Gel filtration of MBP-TNFα-ICD$_{(1-39)}$ was repeated analyzing the protein elution this time by absorbance at 280 nm (black trace), 370 nm (blue trace) and 448 nm (green trace). MBP-TNFα-ICD$_{(1-39)}$ occurred in three different oligomeric states: as a high molecular weight cluster, as dimer binding heme *via* bis-thiolate coordination and as monomer not stably binding heme. For the cluster, absorbance at 422 nm was higher than absorbance at 448 nm (the wavelength characteristic for absorbance of bis-thiolate coordinated heme) indicating that heme binding of the cluster significantly differ from that of the dimer. **g** Elution of MBP-TNFα-ICD$_{(1-39)}$ between 11.0 ml and 17.8 ml from the Superose 6 column was analyzed by SDS-PAGE. **h** Hemin reconstituted MBP-TNFα-ICD$_{(1-34)}$ (compare Fig. 3J) was analyzed by gel filtration on a Superose 6 Increase column. Elution was followed by absorbance at 280 nm (black trace), 370 nm (blue trace) and 422 nm (red trace). Heme cofactor was not tightly bound by MBP-TNFα-ICD$_{(1-34)}$ and lost during gel filtration (and was most likely attached to the column material). Elution volume of MBP (a monomeric protein with a molecular weight of 44.7 kDa when expressed using empty pMalC5X vector) was 17.27 ml (not shown) indicating that MBP-TNFα-ICD$_{(1-34)}$ (with a molecular weight of 46.2 kDa) eluting at 17.09 ml is also monomeric.

i Elution of MBP-TNFα-ICD$_{(1-34)}$ between 10.6 ml and 18.2 ml from the Superose 6 column was analyzed by SDS-PAGE.

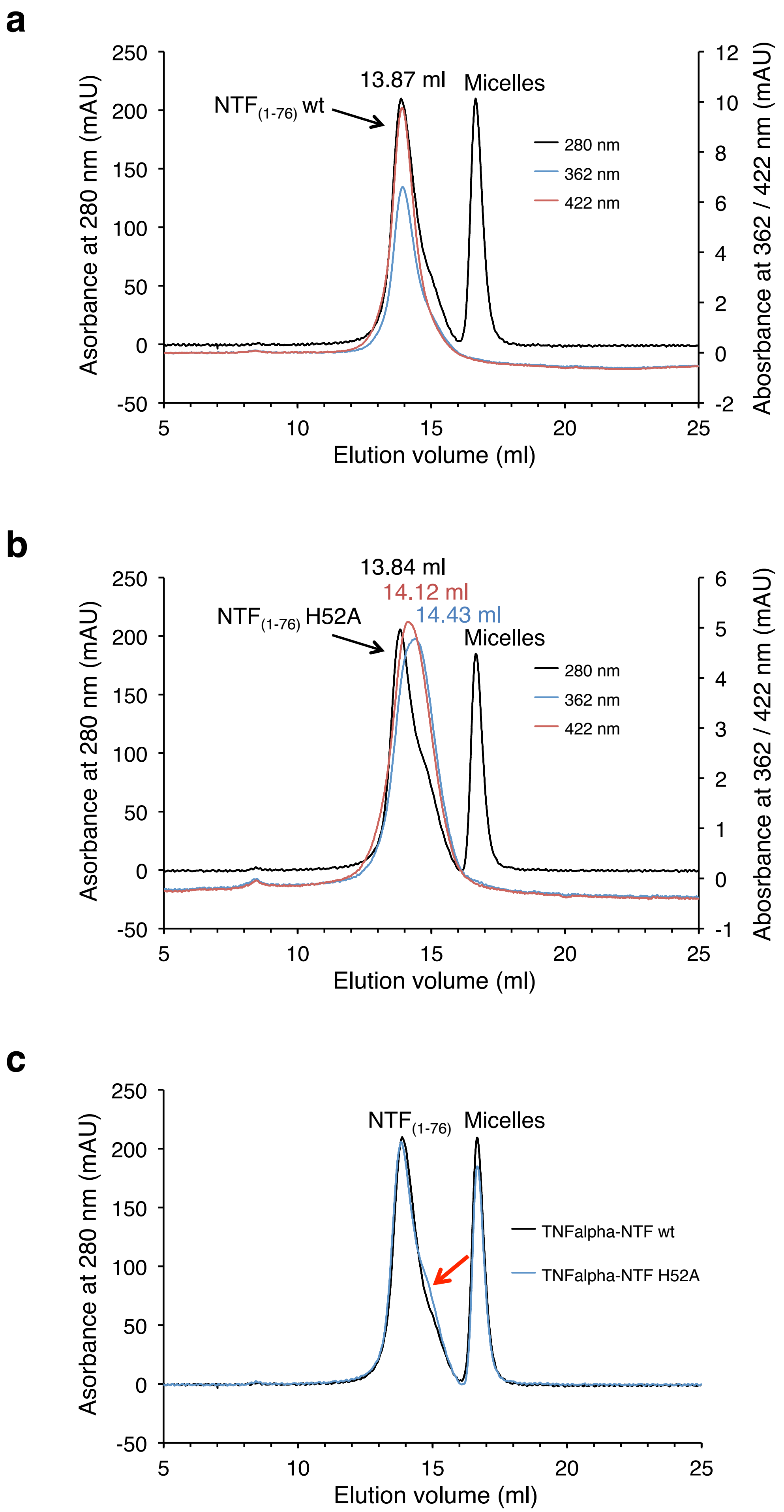
a
13.87 ml
Micelles
NTF(1-76) wt
280 nm
362 nm
422 nm
Asorbance at 280 nm (mAU)
Abosrbance at 362 / 422 nm (mAU)
Elution volume (ml)
b
13.84 ml
14.12 ml
14.43 ml
NTF(1-76) H52A
Micelles
280 nm
362 nm
422 nm
Asorbance at 280 nm (mAU)
Abosrbance at 362 / 422 nm (mAU)
Elution volume (ml)
c
NTF(1-76)
Micelles
TNFalpha-NTF wt
TNFalpha-NTF H52A
Asorbance at 280 nm (mAU)
Elution volume (ml)

Supplementary Figure 4. Gel filtration of MBP-TNFα-NTF$_{(1\text{-}76)}$ wt and H52A. a Purified MBP-TNFα-NTF$_{(1\text{-}76)}$ wt and **b** MBP-TNFα-NTF$_{(1\text{-}76)}$ H52A were analyzed by gel filtration on a Superose 6 Increase column. Elution was followed by absorbance at 280 nm (black trace), 362 nm (blue trace) and 422 nm (red trace). For MBP-TNFα-NTF$_{(1\text{-}76)}$ H52A, the protein fraction binding the heme cofactor eluted later than observed for wt protein. Moreover, the absorbance maxima at 362 nm and 422 nm are present at different elution volumes (14.43 *vs* 14.12 ml), indicating that heme binding is disturbed for this mutant. **c** Comparison of gel filtration chromatograms (absorbance at 280 nm) shows that the MBP-TNFα-NTF$_{(1\text{-}76)}$ H52A protein peak has a broader shoulder than the wt peak (indicated by a red arrow and confirmed in a second, not shown experiment), indicating that the H52A mutation is changing the structure of the TNFα-NTF trimer.

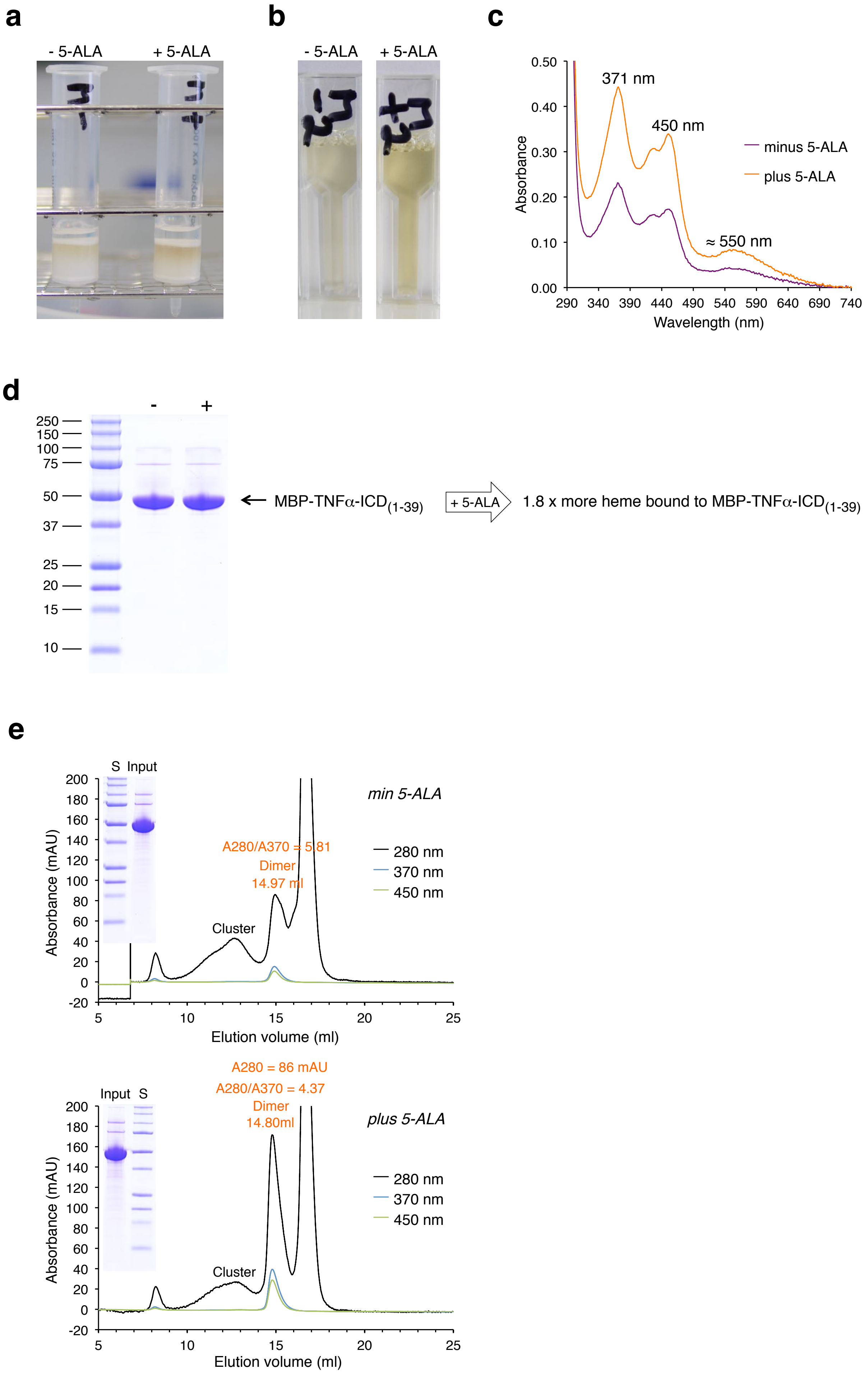

a
- 5-ALA
+ 5-ALA
b
- 5-ALA
+ 5-ALA
c
0.50
0.40
0.30
0.20
0.10
0.00
Absorbance
371 nm
450 nm
≈ 550 nm
minus 5-ALA
plus 5-ALA
290 340 390 440 490 540 590 640 690 740
Wavelength (nm)
d
-
+
250
150
100
75
50
37
25
20
15
10
MBP-TNFα-ICD(1-39)
+ 5-ALA
1.8 x more heme bound to MBP-TNFα-ICD(1-39)
e
S Input
min 5-ALA
A280/A370 = 5.81
Dimer
14.97 ml
Cluster
280 nm
370 nm
450 nm
Absorbance (mAU)
200 180 160 140 120 100 80 60 40 20 0 -20
5 10 15 20 25
Elution volume (ml)
A280 = 86 mAU
A280/A370 = 4.37
Dimer
14.80ml
Input S
plus 5-ALA
Cluster
280 nm
370 nm
450 nm
Absorbance (mAU)
200 180 160 140 120 100 80 60 40 20 0 -20
5 10 15 20 25
Elution volume (ml)

f

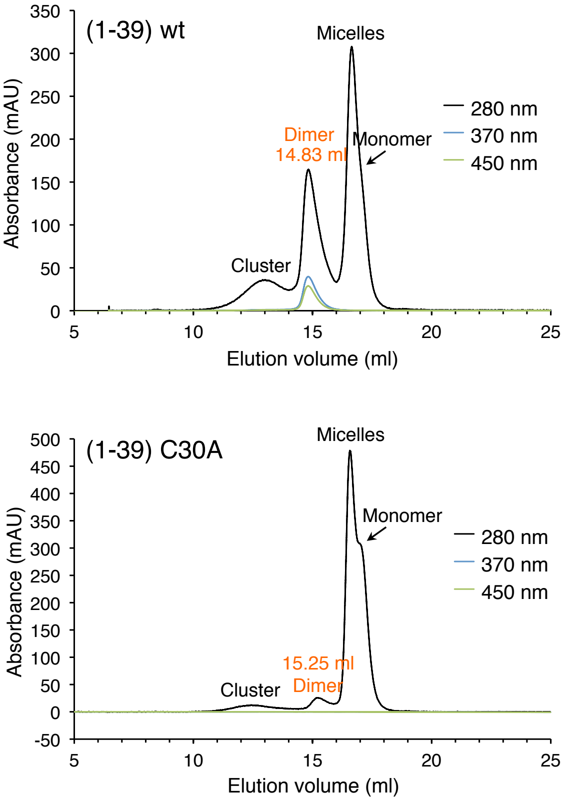
(1-39) wt
Micelles
Dimer
14.83 ml
Monomer
Cluster
280 nm
370 nm
450 nm
Absorbance (mAU)
Elution volume (ml)
(1-39) C30A
Micelles
Monomer
15.25 ml
Dimer
Cluster
280 nm
370 nm
450 nm
Absorbance (mAU)
Elution volume (ml)

g

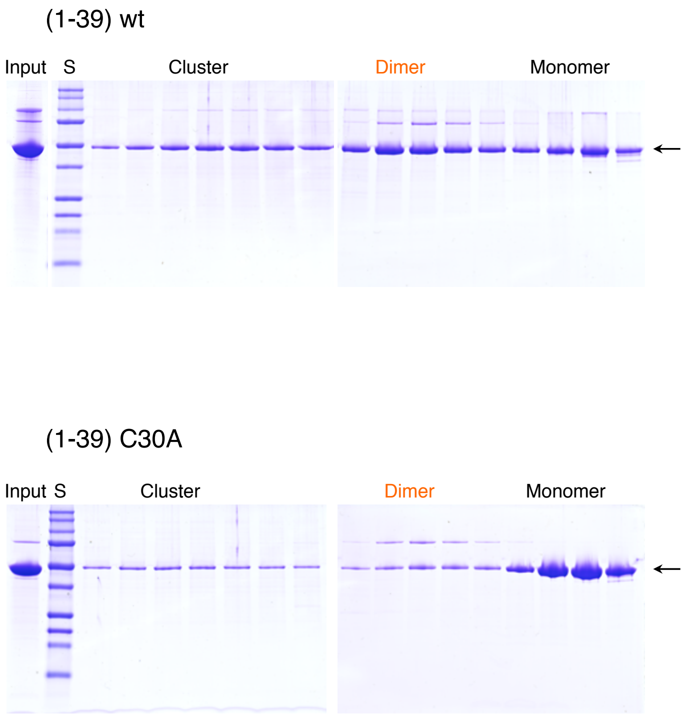
(1-39) wt
Input
S
Cluster
Dimer
Monomer
(1-39) C30A
Input
S
Cluster
Dimer
Monomer

h

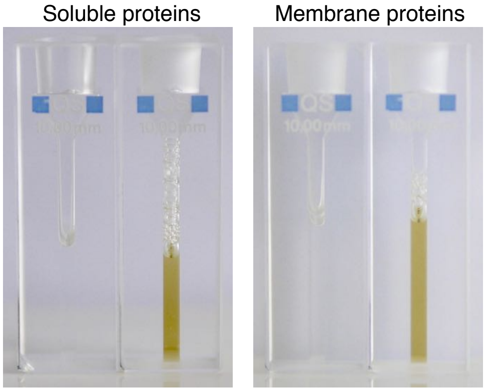
Soluble proteins
Membrane proteins

i

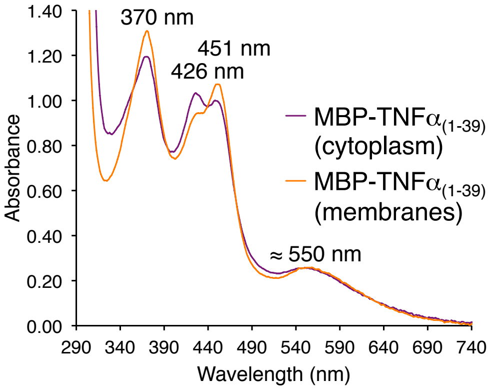
370 nm
451 nm
426 nm
≈ 550 nm
MBP-TNFα(1-39) (cytoplasm)
MBP-TNFα(1-39) (membranes)
Absorbance
Wavelength (nm)

Supplementary Figure 5. Further characterization of heme binding by TNFα-ICD$_{(1-39)}$.

a Membrane bound MBP-TNFα-ICD$_{(1-39)}$ was purified from *E. coli* cells grown in absence (-5-ALA) or presence of 0.6 mM heme precursor δ-aminolevulinic acid hydrochloride (+ 5-ALA) by amylose affinity chromatography. Amylose columns are shown after loading the protein samples and washing with equilibration buffer. **b** Eluted proteins from the amylose columns and **c** UV/VIS spectra of eluted proteins (violet trace, minus 5-ALA, orange trace, plus 5-ALA). **d** Eluted MBP-TNFα-ICD$_{(1-39)}$ proteins purified from *E. coli* cells which had been cultured either in absence (-) or in presence of δ-aminolevulinic acid hydrochloride (+) were analzed by SDS-PAGE. Quantification of the protein bands by ImageJ [1] and comparison with the UV/VIS spectra showed that addition of 0.6 mM δ-aminolevulinic acid hydrochloride to the growth medium increased heme incorporation by about twofold. **e** Membrane bound MBP-TNFα-ICD$_{(1-39)}$ purified from *E. coli* cells grown (this time at 37 °C and induced with 400 μM IPTG) in absence (- 5-ALA) or presence of 0.6 mM heme precursor δ-aminolevulinic acid hydrochloride (+ 5-ALA) was analyzed by gel filtration on a Superose 6 Increase column. Elution was followed by absorbance at 280 nm (black trace), 370 nm (blue trace), and 450 nm (green trace). In presence of δ-aminolevulinic acid hydrochloride dimer content was increased about twofold and cluster content was reduced. Proteins used for gel filtration (inputs) were analyzed by SDS-PAGE [insets in e; for molecular weights of the used standard proteins (S), compare d]. **f** Purified membrane bound MBP-TNFα-ICD$_{(1-39)}$ wt and C30A proteins, respectively, were analyzed by gel filtration on a Superose 6 Increase column. Elution was followed by absorbance at 280 nm (black trace), 370 nm (blue trace), and 450 nm (green trace) and **g** analysis by SDS-PAGE [for molecular weights of the used standard proteins (S) compare d]. **h** MBP-TNFα-ICD$_{(1-39)}$ was purified both, from the cytoplasmic and the membrane fraction. Proteins eluted from amylose columns were concentrated with Vivaspin 500 columns and yellow-green color of both proteins is clearly visible in comparison with buffer containing no protein. **i** UV/VIS spectra of MBP-TNFα-ICD$_{(1-39)}$ purified from the cytoplasmic fraction (containing no Triton X100, violet trace) and the membrane fraction (containing 0.1 % Triton X100, orange trace), respectively. The absorbance maximum at 426 nm of soluble MBP-

TNFα-ICD$_{(1\text{-}39)}$ is likely related to cluster formation in absence of detergent (compare Supplementary Figure 3).

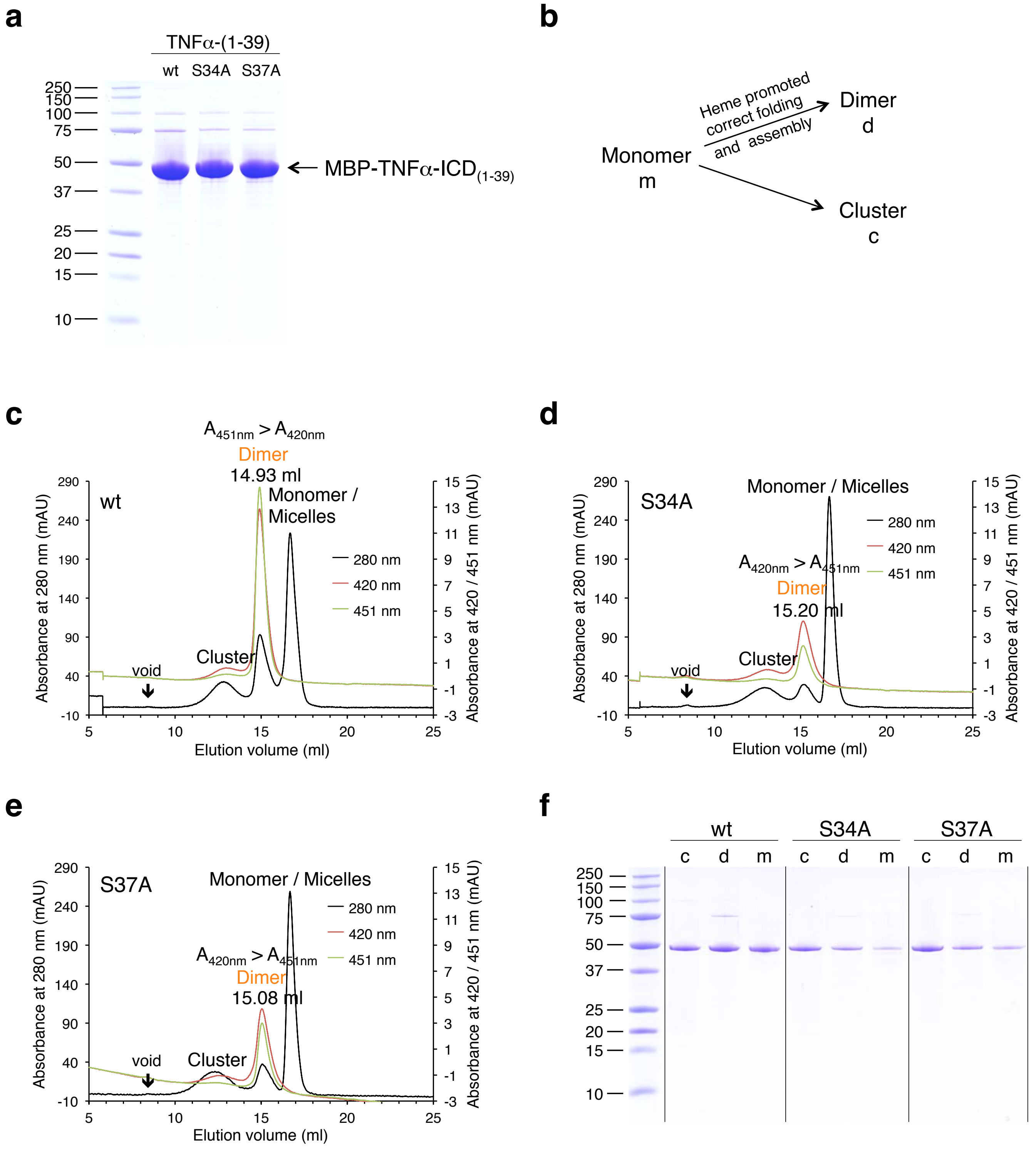

g

	Dimer / Cluster	Dimer / c + d + m	Cluster / c + d + m
wt	1.176	0.365	0.311
S34A	0.527	0.297	0.564
S37A	0.485	0.269	0.555

Supplementary Figure 6. The SXXS dimerization motif within the TMD of TNFα. a Purification of membrane bound MBP-TNFα-ICD$_{(1-39)}$ wt, S34A and S37A proteins by amylose affinity chromatography was analyzed by SDS-PAGE. **b** Pathway for folding of TNFα-ICD$_{(1-39)}$ proposing that correct folding and assembly leads to dimer formation, whereas cluster not dissociated by Triton X100 are likely formed because of overexpression artefacts. **c** Amylose purified MBP-TNFα-ICD$_{(1-39)}$ wt, **d** S34A and **e** S37A proteins were analyzed by gel filtration on a Superose 6 Increase column. Elution was followed by absorbance at 280 nm (black trace), 420 nm (red trace), and at 451 nm (green trace). For the mutant proteins absorbance of the dimer was higher at 420 nm than at 451 nm, indicating changed structure of the mutant dimer or conversion of dimers to cluster after elution from the gel filtration column. **f** Peak fractions [cluster, c; dimer, d; monomer; m] from the gel filtration experiments were pooled, analyzed by SDS-PAGE and then quantified with ImageJ[1]. **g** Using this quantification the dimer to cluster, dimer to total protein and cluster to total protein ratios were determined. In a second, independent experiment similar results were obtained.

a

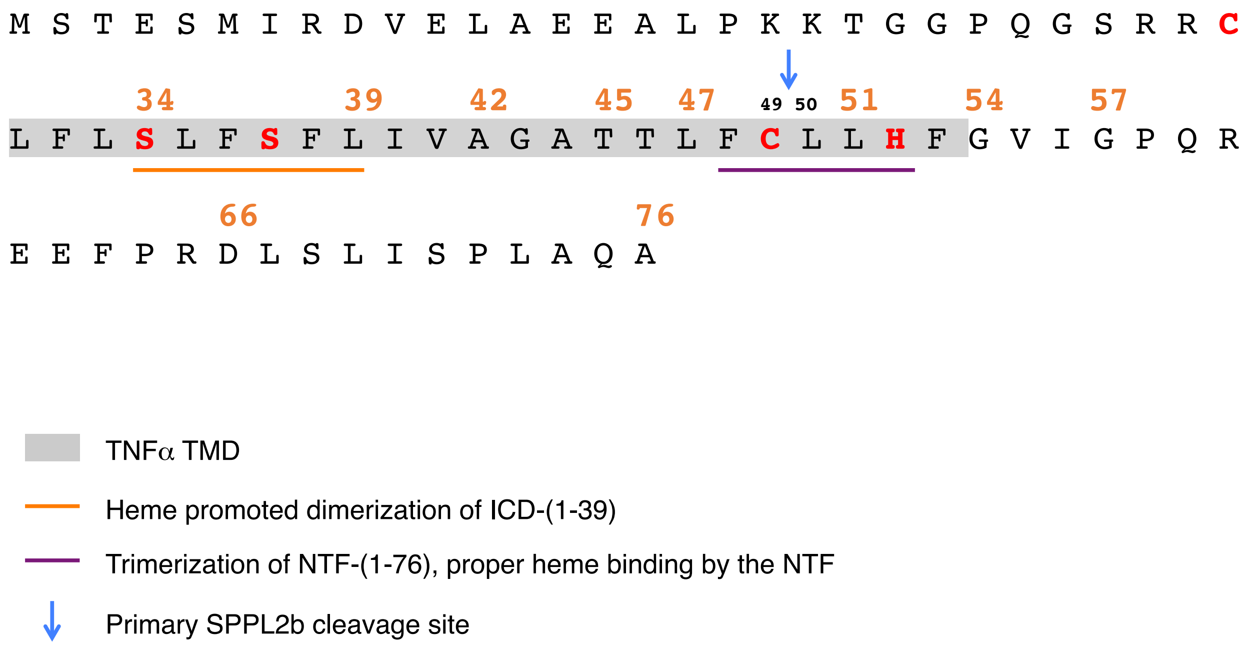

M S T E S M I R D V E L A E E A L P K K T G G P Q G S R R C
34 39 42 45 47 49 50 51 54 57
L F L S L F S F L I V A G A T T L F C L L H F G V I G P Q R
66 76
E E F P R D L S L I S P L A Q A
TNFα TMD
Heme promoted dimerization of ICD-(1-39)
Trimerization of NTF-(1-76), proper heme binding by the NTF
Primary SPPL2b cleavage site

b

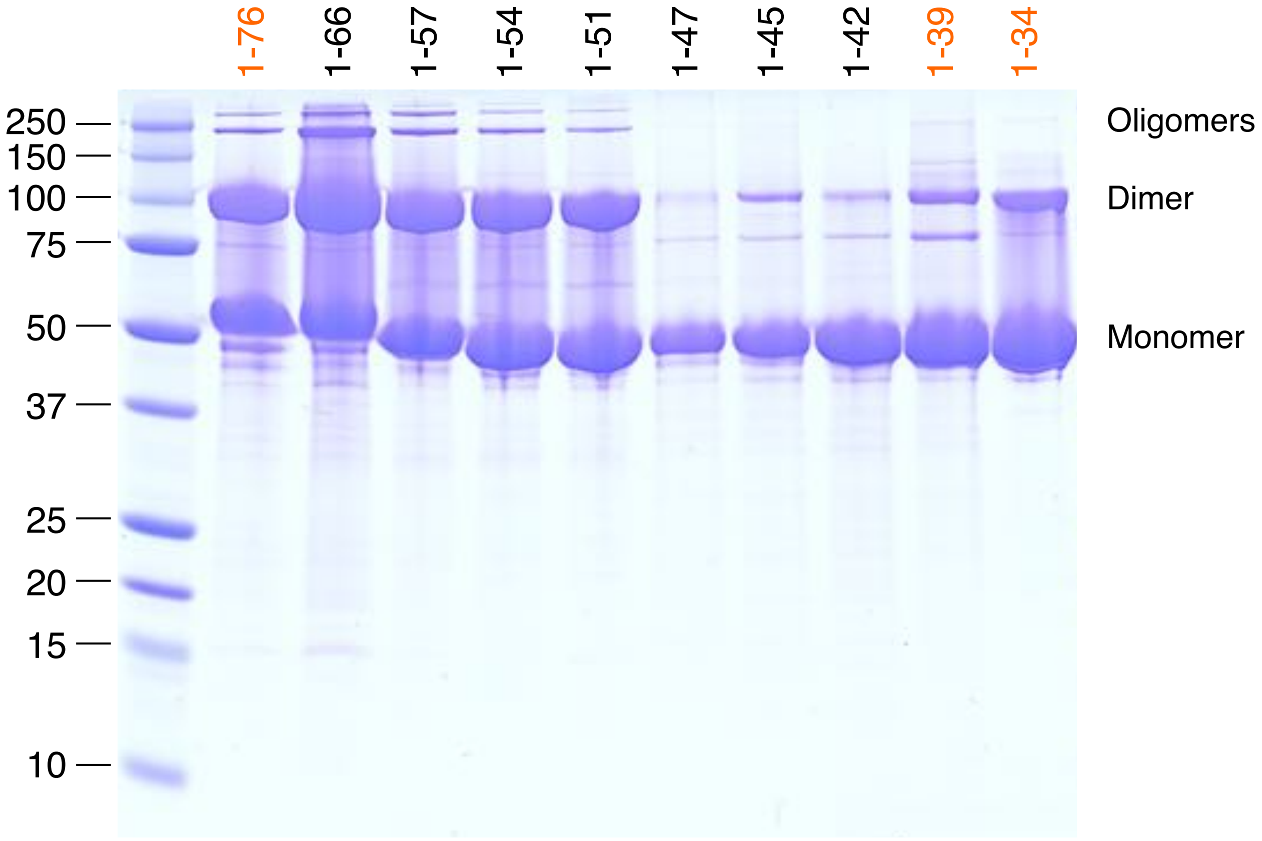

1-76
1-66
1-57
1-54
1-51
1-47
1-45
1-42
1-39
1-34
250
150
100
75
50
37
25
20
15
10
Oligomers
Dimer
Monomer

c

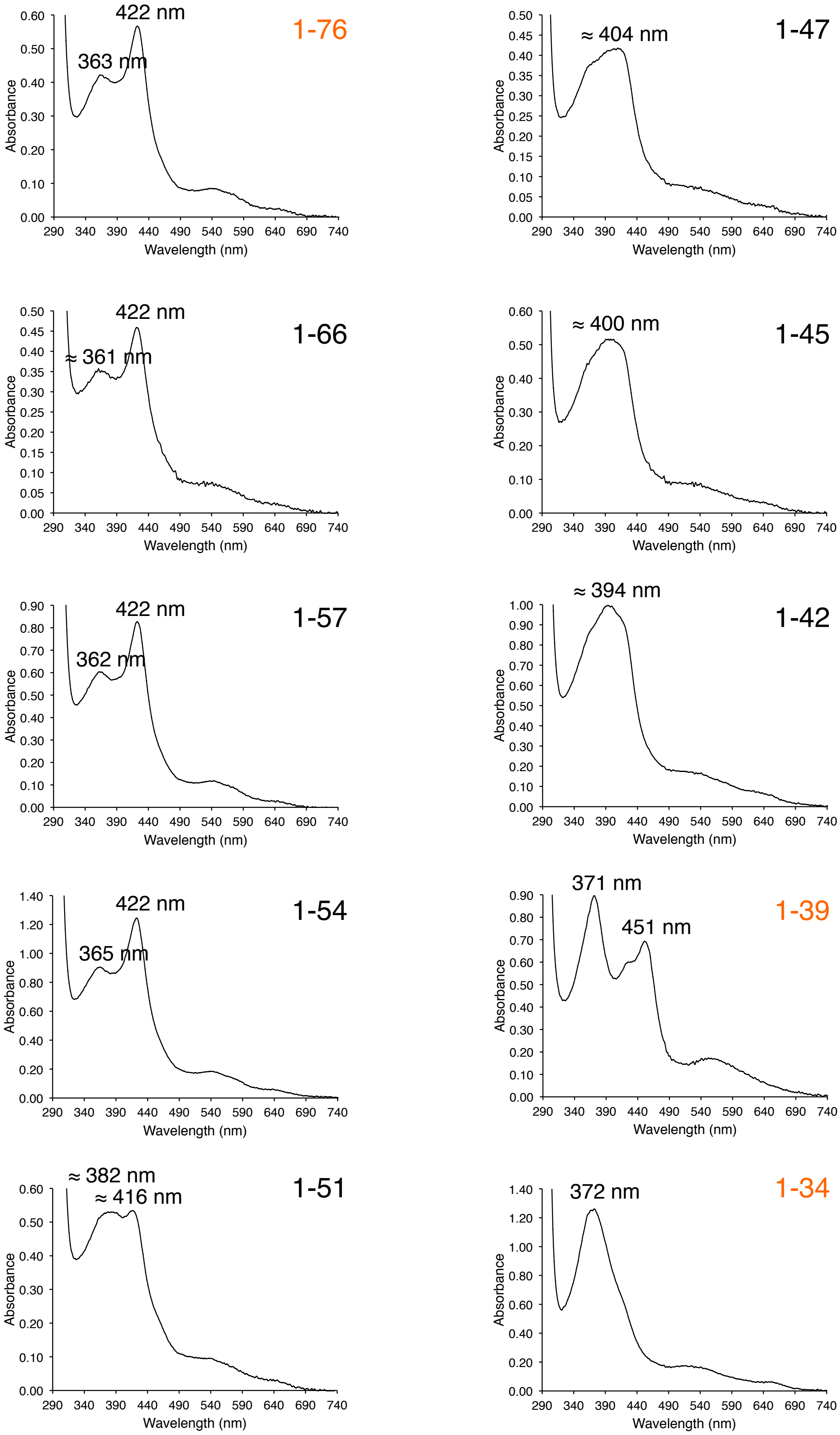

422 nm
363 nm
1-76
≈ 404 nm
1-47
422 nm
≈ 361 nm
1-66
≈ 400 nm
1-45
422 nm
362 nm
1-57
≈ 394 nm
1-42
422 nm
365 nm
1-54
371 nm
451 nm
1-39
≈ 382 nm
≈ 416 nm
1-51
372 nm
1-34
Absorbance
Wavelength (nm)
290 340 390 440 490 540 590 640 690 740

d

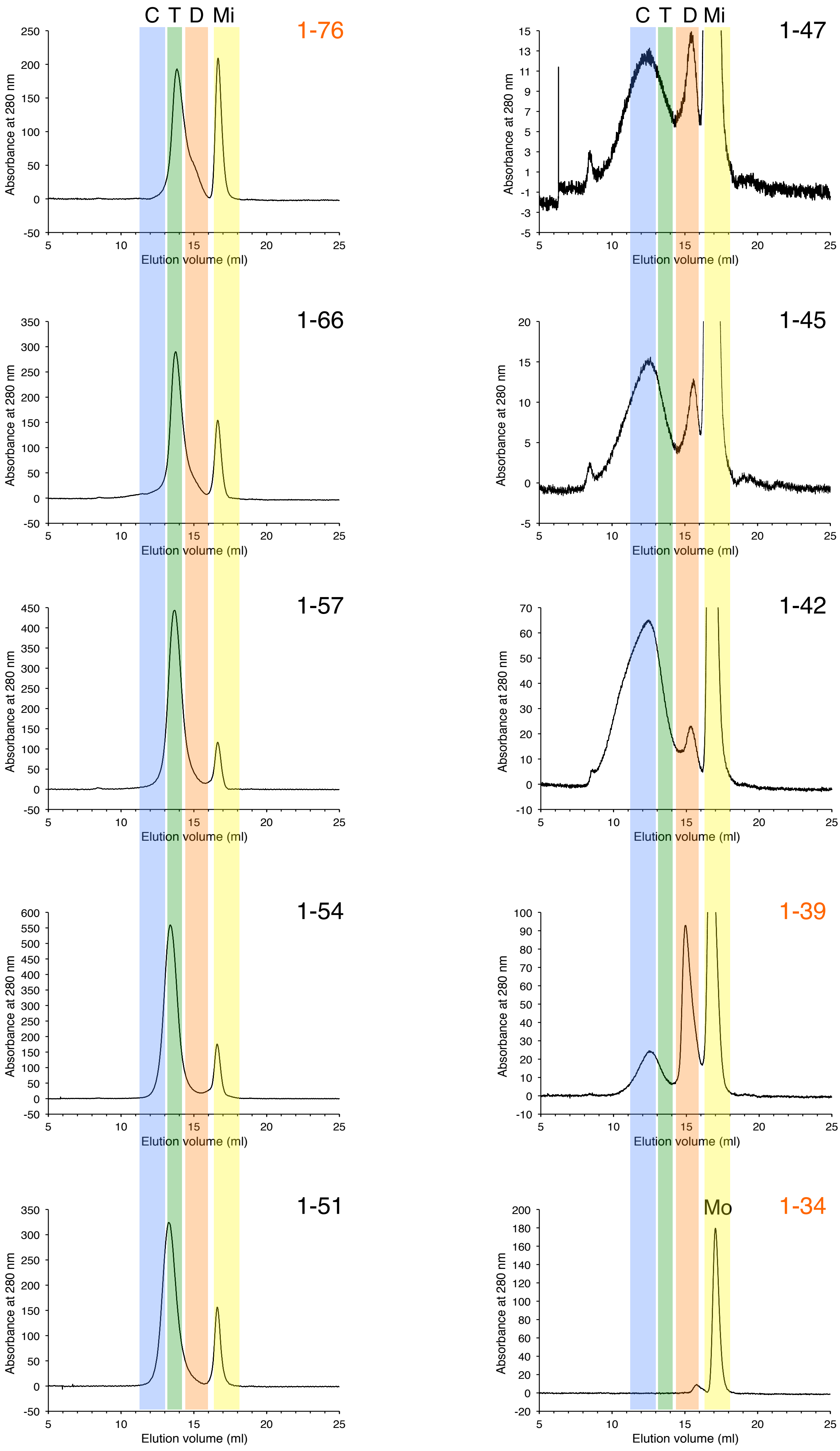

C
T
D
Mi
1-76
1-66
1-57
1-54
1-51
1-47
1-45
1-42
1-39
1-34
Mo
Absorbance at 280 nm
Elution volume (ml)

Supplementary Figure 7. The motif Phe^{48}-Cys^{49}-Leu^{50}-Leu^{51}-His^{52} of TNFα-TMD is important for trimerization and heme ligand switching. a The TNFα-fragments (1-76, NTF), (1-66), (1-57), (1-54), (1-51), (1-47), (1-45), (1-42), (1-39, ICD) and (1-34, ICD) were purified and analyzed by **b** SDS-PAGE, **c** UV-VIS spectroscopy (for ICD-(1-34) the spectrum of hemin reconstituted protein already shown in Fig. 3j is included) and **d** gel filtration on a Superose 6 Increase column [elution volume of cluster, *C*, is indicated in *blue*, of trimeric TNFα proteins, *T*, in *green*, of dimeric proteins, *D*, in *orange* and of micelles, *Mi*, (and monomers, *Mo*, for the both ICDs) in *yellow*].

Supplementary Table 1: Plasmids used in this study

Plasmid name	Description
pTK771	pETDuet-1-*CD74-TMD-(25-64)-wt-10xHis*
pTK816	pETDuet-1-*CD74-TMD-(25-64)-C28A-10xHis*
pTK846	pETDuet-1-*10xHis-CD74-NTF-(1-82)*
pTK871	pMalC5X-*CD74-ICD-(1-42)-wt*
pTK880	pMalC5X-*CD74-(1-216)*
pTK888	pMalC5X-*CD74-ICD-(1-42)-C28A*
pTK900	pMalC5X-*CD74-NTF-(1-81)-wt*
pTK903	pMalC5X-*CD74-NTF-(1-81)-C28A*
pTK952	pMalC5X-*TNFα-NTF-(1-76)-wt*
pTK954	pMalC5X-*TNFα-ICD-(1-39)-wt*
pTK958	pMalC5X-*TNFα-ICD-(1-34)*
pTK960	pMalC5X-*TNFα-(1-233)*
pTK970	pMalC5X-*TNFα-ICD-(1-39)-C30A*
pTK972	pMalC5X-*TNFα-NTF-(1-76)-H52A*
pTK975	pMalC5X-*TNFα-NTF-(1-76)-C30A*
pTK977	pMalC5X-*TNFα-NTF-(1-76)-C49A*
pTK982	pMalC5X-*TNFα-ICD-(1-39)-S34A*
pTK984	pMalC5X-*TNFα-ICD-(1-39)-S37A*
pTK985	pMalC5X-*TNFα-NTF-(1-66)*
pTK987	pMalC5X-*TNFα-NTF-(1-45)*
pTK993	pMalC5X-*Tfr1-NTF-(1-100)-wt*
pTK996	pMalC5X-*Bri2-NTF-(1-101)*
pTK998	pMalC5X-*FasL-TMD-(71-113)*
pTK1006	pMalC5X-*TNFα-NTF-(1-42)*
pTK1008	pMalC5X-*TNFα-NTF-(1-47)*
pTK1014	pMalC5X-*TNFα-NTF-(1-51)*
pTK1015	pMalC5X-*TNFα-NTF-(1-54)*
pTK1016	pMalC5X-*TNFα-NTF-(1-57)*
pTK1022	pMalC5X-*TNFα-ICD-(1-39)-L31P*
pTK1023	pMalC5X-*LOX-1-NTF-(1-88)*
pTK1029	pMalC5X-*Tfr1-NTF-(1-100)-C62A*
pTK1030	pMalC5X-*Tfr1-NTF-(1-100)-C67A*

DNA sequences of these constructs are listed in Supplementary Data file 1.

Supplementary Note

Triton X100 was used as detergent for all experiments with MBP membrane fusion proteins [including TNFα-ICD$_{(1-39)}$ proteins] shown in the Supplementary Figures.

Supplementary Reference

1. Schneider, C.A., Rasband, W.S. & Eliceiri, K.W. NIH Image to ImageJ: 25 years of image analysis. *Nat Methods* **9**, 671-5 (2012).
