# Supplementary material for: Heme binding of transmembrane signaling proteins undergoing regulated intramembrane proteolysis
Source: Commun Biol. 2020 Feb 14;3:73. doi: 10.1038/s42003-020-0800-0 (PMC7021776; doi:10.1038/s42003-020-0800-0)
Supplement: Supplementary file 2 — Description of Additional Supplementary Files [file 42003_2020_800_MOESM2_ESM.pdf]

Description of Additional Supplementary Items

Supplementary Data 1. DNA sequences of cloned fragments [*Nco*I/*Hind*III and *Nco*I/*Xho*I inserts of pETDuet-1 and *Sac*I/*Eco*RI inserts of pMalC5X] are shown in this file.
